# Evolution in Dentistry

**Published:** 1890-05

**Authors:** J. A. Robinson


### THE DENTAL REGISTER.

VOL. XLIV.] MAY, 1890. [No. 5.

### COMMUNICATIONS.

### Evolution in Dentistry.

BY J. A. ROBINSON, D.D.S.

If dentistry is to become a professional fact in the highest sense, a profession with the highest aims, it must become so through the laws of evolution. The first thing to be accepted and thoroughly understood is the law of *physical* evolution, for that is the base of spiritual evolution, and all the professions are based on that fact. There can be no profession without the love of our neighbor as ourselves, and that is the spiritual law, and physical evolution is the alphabet and the school master to teach us, and show us the way. The mechanics and conscience are only the tools we use to carry forward these higher aims. As the germ or the protoplasm is the starting point of all life, and that is endowed with the power of progress, so dentistry began with the doctor of medicine, and it is in perfect accord with the law of evolution that in the end the dentist should be the doctor of dental surgery. He came, in fact, from this common stem of *cure*, and he was not a typical representative but a connecting link of what was to become flower-bearing and fruit-bearing; a common trunk of the learned professions. The change has been as great and the differentiation as marked and the progress as certain as that exhibited in our civilization between the log hut of the original back woods settler who first came to this western wilderness and the palatial residence of the millionaire of to-day.

There are certain individuals who have advanced to the dignity of scientists and men of letters in our profession, and others who keep working on a level plane to the end of life. Go work in the vineyard is the law of the New Testament and the law of life, and not like Thoreau, who, when he had made what he considered a perfect lead pencil threw away his tools and abandoned his shop and refused to make another, but lived a nomadic life and communed with woodchucks, blue birds and sparrows.

The transmutation of rubber by vulcanization made as great a change in the practice of dentistry as there is in nature between the typical fishes and the true reptile, and the real practice in artificial dentures stood as widely apart as the reptile and the bird life. Good teeth, by the use of sulphuric ether in 1839 and 1840, were extracted by the bushel to make way for what was advertised to be the *cure-all* for those who had by neglect or heredity been victims of toothache or loss of teeth. So great was the mania for tooth extraction that offices were fitted up in Boston where persons were employed for nothing else but extracting teeth with sulpuric ether "without pain," and the first vulcanizers that were made held two gallons of water, and had capacity for vulcanizing twenty sets of teeth at a single heating. Gilbert's air chamber, or central cavity, came along about 1839 or 1840, and was made with a piece of hard leather the size and shape wanted; the leather was placed in a zinc model after the gold or silver plate had been swaged. This piece of leather was placed on the zinc die under the plate, the die and plate placed in the lead counter die and struck with a heavy hammer sufficiently to produce a vacuum or depression, that assisted greatly in holding the plate in place while talking or eating, and this little contrivance was the first invention in dental art that was patented. Between the years 1839 and 1842 was a remarkable epoch in the improvement of dental art; it was not confined to one thing, but a general uplifting or going forward, an evolution of the whole line of practice. Spooner had recommended arsenic combined with morphine to devitalize the pulps of the teeth and Dr. S. P. Hullihen, of Wheeling, W. Va., had published his method of immediate filling where the pulp was exposed, and then by drilling a small hole through the alveolar process to meet the apex of the root for discharge of blood and prevent inflammation and leave the tooth to get well by a natural process. I

have no way of telling the exact data of this upward process, as I write entirely from memory of what took place fifty years ago.

In cases of acute inflammation and suppuration the remedies were very few and simple, and consisted of lancing the gums above the offending tooth and giving saponaceous pills made of soap combined with aloes as a stimulating purgative to change the secretions and recommended patience and rest. This treatment was recommended after the pivot-teeth had been inserted on a wooden dowel, to prevent taking cold, and having a swollen face. We had not learned to stop the foramen of the tooth to prevent the air being forced through to create inflammation and produce a swollen face, but laid all the trouble to "taking cold" during the operation. Dr. Hullihen also recommended drastic cathartic pills after the operation and then drilling a small hole to meet the foramen in immediate tooth-filling. For hemorrhage after tooth extraction we used a small vial cork, to cork the opening as we would do with a small bottle. These were simple natural remedies and were suggested like all remedies—the difficulties suggested the remedies. I think this was before Monsel's salts had been discovered for hemorrhage.

Not one of the least evidences of evolution in our profession are those of *personal friendships*; to find the contrast we must look back fifty years.

To those persons who have entered the profession through dental colleges there can be no adequate description of the feelings of envy and jealousy that existed between good dentists at that time. Every dental office was a sealed enclosure. There was no bow of recognition or hand-shaking on the street, and if a dentist entered the office of any other dentist he was met at the door with a look of "What do you want here?" that forbade his calling the second time. The dentist had only reached the productive and reproductive and muscular stages of development in professional life, and personal friendships were separated as widely as the combination and non-combination dealers, or the mollusks from the vertebrates. Even as late as 1853, when I first came to Cleveland and suggested forming a city society, in canvassing around among the select few who were more intimate as to whom we should invite some dentist would ask, "Shall you invite Old Hog-Eye,?" alluding to one who had a defect in one of his eyes; and another would remark, "I hope you will not let that Irishman come in;" and another would say, "We don't want any thing to do with Old Wright." Dr. Wright was working hard to introduce porcelain plates, and almost every dentist had some degrading nickname, that he was contemptuously called, and personal innuendos about the neighboring dentist were far more frequent than is heard to-day.

Atkinson was at the meeting, and with his big heart he took them all in, and better feeling prevailed, and finally there was a general invitation to all the dentists in the city. I was particularly struck with the evolution of sentiment while attending a dental meeting in Buffalo, about two years ago, and then again in Cleveland, last October, at the Ohio State Society meeting. The second forenoon was devoted to eulogizing Dr. F. H. Rehwinkle, of Chilicothe; Dr. A. Berry and Dr. C. R. Taft, of Cincinnati, and Dr. R. L. Evans, of Toledo, former members of the State Society who had died during the past year. Prof. Taft was at his best in his oration on Dr. Rehwinkle, as being the truest in thought, the bravest in speech for justice and right; the pure soul of honor without a particle of dross throughout his whole intercourse of society and his family, and a serene faith in trusting our profession on its merits to take care of the future as well as the present by its intrinsic worth; that no act of his needed an apology, but his whole life, like pure truth, belonged to the present and to all time. When Dr. Harroon, of Toledo, rose to speak to the memory of Dr. C. R. Taft, of Cincinnati, the tears blinded up his large blue eyes until they fell down upon his long gray beard, and with choked utterances he gave a loving tribute to the personal worth of a worthy worker in the profession, and Dr. H. A. Smith spoke with the tenderest feeling of the worth of those who had passed away within the year. It takes something more than professional etiquette to do this. Life is carried to higher and higher points until it gives the emotions of the heart which is the foundation of the Christian religion, and the cap-stone and seal of our common brotherhood in all the professions. Self-esteem is healthful; it strengthens character and helps to elevate society, but when our co-laborers are held in disesteem simply because they are in humble life or can not *put on style,* it is hateful and destroys that brotherly love which is the bond of professional life. While in Cleveland, in 1854, I had a patient from an adjoining town call at the office with some teeth to be filled. On examination I found the most prefect fillings in his mouth I had ever seen. I called my brother's attention to the work and learned it was done by Corydon Palmer, of Warren, Ohio. I said to my brother "This work has taken all the conceit out of me. I must see this Corydon Palmer. I thought I was the best operator in the West but I am not." So the next Saturday I took the train and went to spend Sunday with Palmer. I found him a very unpretentious person but thoroughly in love with his profession. We talked almost all night and all next day Sunday, and I came home saying I had found the best dentist I had ever seen, in an obscure town in Ohio, who was so modest and so unpretending that he did not know himself how much he knew or how well he could work. Shortly after, with repeated invitations, he came and made me a visit of three or four days. We made instruments and talked all sorts of things that ought to be done among the dentists and had a real *love feast.* That was the beginning of a pleasant, lasting and confiding friendship.

---

ARTIFICIAL TONGUE.—Dr. Poncet, of Lyons, has invented an artificial tongue for improving articulation in cases where that organ has been extirpated. The apparatus consists of a "pocket" of soft rubber containing fluid, and joined on to a plate which is fixed to the lower teeth. "A patient wearing this apparatus can eat and speak quite satisfactorily, and there is no dribbling of saliva, the latter being swallowed as in health." —*Edinburgh Med. Journal,* Feb., 1890.

---

DR. O. W. HOLMES says it is not true. The poet-physician has had it brought to his notice that a learned small girl of Boston has spoken of him as having been for many years a professor of monotony at Harvard University.

